# Editorial: Learning identities in times of COVID-19

**DOI:** 10.3389/fpsyg.2023.1251160

**Published:** 2023-07-25

**Authors:** Kateřina Juklová, Ana-Maria Cazan, Camelia Truţa

**Affiliations:** ^1^Department of Pedagogy and Psychology, Faculty of Education, University of Hradec Králové, Hradec Králové, Czechia; ^2^Faculty of Psychology and Education Sciences, Department of Psychology and Education Sciences, Transilvania University of Braşov, Braşov, Romania

**Keywords:** learning identities, learning experience, COVID-19, learning processes, learning outcomes

## 1. Our questions

As part of this series, we were interested in the experiences that the COVID-19 pandemic has brought and their relevance to learning processes and outcomes. We were interested in understanding how students, teachers, and even parents related to themselves as learning agents in the context of online or hybrid education and how their expectations and attitudes varied during the pandemic. We anticipated that these were not necessarily only changes in a negative sense, but also changes in a positive direction, new opportunities. The questions we asked, therefore, were: Have the effects of the COVID-19 pandemic on learning been felt only in a negative sense or also in a positive sense? In what ways can lessons be learned from this new learning experience?

## 2. What has been explored and how?

The papers included in this series have provided empirical findings on learning experiences during the COVID-19 pandemic from five countries (China – Wang et al.; Yu et al.; Zhang et al.; Romania – Stan et al.; Austria – Paechter et al.; Spain – Sanz and López-Iñesta, and Algeria – Yahiaoui et al.). With the exception of one, which had a mixed research design (Yahiaoui et al.), these are quantitative research studies exploring the effects of this new experience on learning processes and outcomes. The majority of the studied experiences came from university students, but in one case the participants were university students and teachers (Yu et al.), and in another study, they were parents of middle school students. The size of the research samples ranged from research population from a single institution of 100–200 participants to huge samples of thousands or hundreds of thousands of participants.

The learning experience during the COVID-19 pandemic was related in most studies to the immediate forced transition to online learning and the suspension of face-to-face contact between students, teachers, and peers, situations for which teachers and schools were not prepared. This learning experience was operationalized in the vast majority of studies through concepts of negative emotionality and predominantly undesirable consequences (uncertainty, stress, anxiety, depression, hyperarousal). An exception is a study operationalizing the consequences of pandemic as environmental factors positively moderating the relationship between the impact of attitudes toward entrepreneurship education on entrepreneurial self-efficacy (Zhang et al.). Also, an article mapping the Algerian learning experience during the pandemic only looks at the benefits of e-learning on motivation and learning outcomes without being tied to the negative effects of this extraordinary experience (Yahiaoui et al.).

The effects of the new experience were examined in several ways. One was a direct inquiry into the subjectively experienced degree of change in various areas of the student's functioning. For example, a study by Wang et al. mapped the subjectively perceived impacts of the pandemic on Chinese students' academic and social activities and the extent to which some negatively experienced emotions or states (anxiety and depression) were present. However, the studies in this series were entirely dominated by exploring impacts in terms of identifying online learning experiences or experienced emotions and relating them to student engagement or academic achievement. In these cases, the authors of the studies often relied on the theory of Triadic Reciprocal Determinism (Bandura, 1978), assuming reciprocal interactions between the (learning) environment, individual characteristics and behavior (e.g. Wang et al.; Zhang et al.). In line with this framework, they assumed that students' learning experiences, including motivational aspects (e.g., perceptions of self-efficacy) with links to learning outcomes, are not only influenced by individual factors or environmental ones but also by the reciprocal interactions between these two factors. Therefore, in the new learning environment forced by the pandemic, students may have different learning experiences, and these new learning conditions may play a mediating role in the relationship between individual and behavioral characteristics.

A number of studies have examined the role of individual or personal characteristics. For example, Wang et al. used a latent class analysis method to distinguish between four groups of students with differently successful rates of adjustment to new conditions manifested in terms of academic and social activities and mental health. The study by Stan et al. explicitly focused on examining the mediating role of self-regulated learning strategies in individuals' processes of adjustment to new learning environments. Similarly, the Paechter et al. study focused on personal dispositions, which they examined as factors directly or indirectly promoting or inhibiting academic performance during the COVID-19 pandemic.

Along with individual characteristics, extracurricular factors were also of research interest. The role of technological and economic factors for academic performance during the pandemic was investigated by the Sanz and López-Iñesta's study and the role of experience and attitudes toward e-learning was investigated by Yahiaoui et al.. Finally, the effect of the field of study on the learning experience during a pandemic from the perspective of students and teachers was researched (Yu et al.).

Cross-sectional research design was often used to identify the relationships between the factors and variables under study and various types of regression analysis, structural equation modeling, latent class analysis, and analysis of variance were employed. Thematic analysis, cluster analysis, and cognitive mapping were used to analyze qualitative data in the case of a mixed research study (Yahiaoui et al.).

## 3. What was found?

The main aspects revealed by the studies included in this series are the following:

- The effects of the pandemic on students varied - depending on their individual characteristics, motivation, self-regulatory skills and support from teachers and parents. The results of a large proportion of our studies suggest the usefulness of differentiation in education in relation to the individuality and needs of different groups of learners and encourage the provision of more targeted pedagogical interventions. For example, the study by Wang et al. showed that there is a non-negligible group of students for whom it is appropriate to address not just learning but their mental health also.

- In at least some areas, the economic situation of students' families played a significant role and, due to uneven access to virtual environments and the need for some students to support their entire families, had a significant impact on academic achievement and was a source of social inequalities. What also needs to be taken into account in education is the overall living situation of students, and those who have to work hard while studying should be provided with special assistance or differentiated support (e.g., Sanz and López-Iñesta).

- In situations of uncertainty, the need for self-regulated skills as structuring mechanisms to control the learning environment increased. These acted as protective factors against the emergence of depression, helplessness and negative academic outcomes. Thus, in line with the results of the Stan et al. study, deliberate ongoing training in self-regulation skills can be considered a good prevention of perceived excessive stress and a potential tool for student resilience. The sub-skills, especially the ability to autonomously structure one's own learning environment and to set individual goals as skills beneficial in a pandemic era, also remain essential for navigating a rapidly changing world. These findings call on educators to place greater emphasis on individualization and personalization in education to promote learner autonomy.

- In general - the benefits of online learning for motivation and learning outcomes were confirmed, but they need to be carefully studied in more differentiated ways, as not only students differ in their abilities, expectations and emotionality, but also teachers differ in the ways in which they use online tools and their modalities (Yu et al.). Along with confirming the benefits of online teaching, it has been shown that its effectiveness depends on by whom, how and with what respect for students' needs it is used, and that face-to-face contact between students and teachers can lead to reduced anxiety and depression under optimal conditions (Wang et al.).

## 4. Unanswered questions

The results of the studies included in this series offered insight into the factors that facilitated or hindered the adaptation to the changed learning environment imposed by the pandemic. Even thou some studies tried to address the immediate impact on learning outcomes such as academic performance (e.g., Stan et al.; Sanz and López-Iñesta), the long-term impact of the changes imposed by the pandemic on specific learning outcomes remains a topic yet to be explored. For example, what are the consequences in terms of acquisition and development of transferable skills? How did students manage to develop and exercise presentation and self-presentation skills, networking skills, leadership, and team-working skills? What role did other educational factors play in the development of such skills and what practices did teachers use for fostering the efficient practice of transferable skills in an online environment?

Another question that still needs to be addressed is how the experiences brought by the changes during the pandemic and the adaptation to these changes shape in the long-term the learning identities of both students and teachers. We now know that individual characteristics, motivation, self-regulatory skills, teacher and parental support, and (economic) access to the virtual environment impact students' and teachers' experiences, engagement, and attitudes toward education. What impact will have these experiences on future attitudes toward learning? How will students who experienced anxiety, depression, and stress approach future changes in the educational system? And how will teachers use the experiences they had in creating better online and offline learning environments for the students? Which of the newly acquired strategies and learned lessons remain in their modes of operation after the return to “normal” and will continue to be viable in the future?

## 5. Conclusion

Exploring learning identities during the pandemic contributed to the expansion of knowledge on the topic and generated new research directions on cognitive, emotional, behavioral, and social dimensions of learning. Despite its disruptive effect, the pandemic not only brought new demands and changes but also contributed to the development of new ways of social organization, new positions, and roles, thus the pandemic imposed the development of a new sense of self in the context of education, shaping the future of learning and teaching (Smith et al., 2022). In line with the results of the studies in this series, we can conclude that these changes developed our resilience and capacity to respond to future challenges (Cain et al., 2022). As such, there are significant findings and implications from these studies, in that they present the reality that teachers and students faced and which led to the improvement of educational processes, to the increase of the wellbeing of all actors, and to the support of their autonomy, motivation, and engagement in education.

## Author contributions

KJ, A-MC, and CT wrote the manuscript. All authors read and approved the submitted version.
